# Health status impairments in tyrosine kinase inhibitor-treated chronic myeloid leukemia patients versus blood or marrow transplant survivors and non-cancer controls

**DOI:** 10.1038/s41375-026-03123-w

**Published:** 2026-08-21

**Authors:** Smita Bhatia, Qingrui Meng, Lindsey Hageman, Sarah B. Palmer, Eunice E. Choe, Nora Balas, Wendy Landier, Omer Jamy, Pankit Vachhani, Alysia Bosworth, F. Lennie Wong, Jorge Cortes, Stephen J. Forman, Daniel J. Weisdorf, Saro H. Armenian, Ravi Bhatia

**Affiliations:** 1 University of Alabama at Birmingham, Birmingham, AL USA; 2 City of Hope, Duarte, CA USA; 3 University of Minnesota, Minneapolis, MN USA

**Keywords:** Quality of life, Epidemiology

## Abstract

Tyrosine kinase inhibitor (TKI) therapy has resulted in a significant reduction in chronic myeloid leukemia (CML)-related mortality. However, the impact of TKIs on the health status of long-term CML survivors remains unstudied. We examined the health status of 62 TKI-treated CML patients who did not receive blood or marrow transplantation (BMT) (CML_TKI; median follow-up: 12.5 y) compared with an unaffected population (303 non-CML controls), and CML patients treated with BMT but without TKI exposure (220 CML_BMT; median follow-up 19.4 y). Participants self-reported general health, functional status, activity limitations, and pre-frailty/frailty. Overall, 71.0% of CML_TKI participants had ≥1 adversely-affected domain vs. 42.6% of non-CML and 65.9% of CML_BMT participants. When compared with the non-CML controls, the CML_TKI cohort was more like to have poor general health (aOR = 4.43, 95%CI = 1.8–10.7), functional impairment (aOR=2.84, 95%CI = 1.4–6.0), and activity limitation (aOR=3.37, 95%CI = 1.7–6.7) and more likely to be frail/pre-frail (aOR=3.71, 95%CI = 1.7–8.1). The CML_TKI and CML_BMT cohorts were comparable with respect to poor general health (aOR = 0.95, 95%CI = 0.5–2.0), functional impairment (aOR = 1.59, 95%CI = 0.8–3.0), and pre-frailty/frailty (aOR = 1.92, 95%CI = 0.92–4.0), but the CML_TKI cohort was more likely to report activity limitation (aOR = 2.2, 95%CI = 1.1–4.5). These findings inform a need for long-term monitoring and development of risk mitigation strategies to improve the health status as a treatment goal in CML care.

## Introduction

An estimated 10,000 individuals are diagnosed with chronic myeloid leukemia (CML) in the United States each year [1]. Use of tyrosine kinase inhibitor (TKI) targeted therapy has resulted in a significant reduction in CML-related mortality and possible cure in a fraction of patients [2, 3]. Since the approval of imatinib as first-line targeted therapy for CML in 2001, five additional BCR::ABL1 BCR-ABL TKIs have been approved by the US Food and Drug Administration (FDA) for first-line and/or subsequent lines of treatment: nilotinib, dasatinib, bosutinib, ponatinib, and asciminib. Although allogeneic blood or marrow transplantation (BMT) remains the most consistently curative treatment for CML, the associated morbidity limits its acceptance, particularly among older patients and those with pre-existing comorbidities [4–6]. Currently, even among younger, relatively healthy patients, TKIs have largely replaced BMT as the treatment of choice due to convenience of administration, and limited early morbidity/mortality, with BMT reserved for patients who have experienced failure of available TKIs or disease progression.

Several studies have reported complications associated with TKIs, including dermatologic, cardiovascular, hepatic, and renal toxicity [7–24]. However, the impact of TKIs on the health status (including general health, functional impairment, activity limitation, and frailty) of long-term CML survivors has not been widely studied [25]. Self-rated general health, a single-item measure, has been reported as an independent predictor of mortality in the general population and cancer patients treated with conventional therapy [26–29]. Functional impairment and activity limitations represent an inability to perform everyday tasks, ranging from basic self-care (like bathing and dressing) to more complex activities (like managing finances or cooking), often stemming from physical or cognitive decline due to aging or underlying health conditions, leading to reduced independence and quality of life. These domains of health status are key indicators of overall health, signaling potential risks for falls, social isolation, hospitalization, and nursing home admission [30]. Frailty is characterized by exhaustion, weakness, low physical activity, slow walking speed, and unintentional weight loss. Frail individuals exhibit three or more of these indices, while pre-frail individuals exhibit two indices [31]. Both pre-frailty and frailty are associated with a higher hazard of mortality in both non-cancer populations [32, 33] and among cancer survivors [31, 34]. Although TKI discontinuation is increasingly becoming a goal of therapy for CML patients, only ~25% of patients achieve treatment-free remission (TFR), and most patients are expected to remain on therapy indefinitely [35]. The reduction in mortality rate following introduction of TKIs for CML treatment has led to an increase in the prevalence of CML in the US from 30,000 in 2000 to nearly 74,000 in 2021 [36]. It is estimated that there are >1,750,000 CML patients globally, and the prevalence is projected to exceed 4 million patients worldwide by 2040–2050 [37, 38]. The current need for long-term, and for most patients, indefinite TKI therapy, raises important questions regarding the impact of the TKIs on the self-reported long-term health status of CML patients, in the context of their sociodemographic and comorbidity profile. We leveraged the BMT Survivor Study (BMTSS) to examine the health status of TKI-treated CML patients compared with two groups of individuals: (i) an unaffected population, and (ii) CML patients treated with BMT but without exposure to TKIs.

## Materials/ subjects and methods

### Study population

BMTSS uses a cohort study design to examine long-term outcomes of individuals who underwent BMT between 1974 and 2014 at the University of Alabama at Birmingham (UAB), City of Hope (COH), or University of Minnesota (UMN) and survived ≥2 years after BMT and two age and sex frequency-matched comparison groups: (i) unaffected siblings of BMT survivors; (ii) individuals with hematologic malignancies treated with non-BMT therapies who had survived ≥2 years after diagnosis of their hematologic malignancy. Participation involved completion of a 255-item BMTSS survey that covers demographic characteristics (race/ethnicity, education, annual household income, and health insurance coverage), chronic health conditions (as diagnosed by the healthcare provider), chronic graft vs. host disease (cGvHD), and health status.

We determined the severity of the chronic health conditions, using the Common Terminology Criteria for Adverse Events (CTCAE) developed through the National Cancer Institute by a multidisciplinary group of experts [39]. This scoring system is intended for use in scoring both acute and chronic conditions in patients with cancer and survivors of all ages [5, 40]. The system grades conditions as mild (grade 1), moderate (grade 2), severe (grade 3), life-threatening or disabling (grade 4), or fatal (grade 5). For our study, a total of 137 health conditions were scored using CTCAE v5.0. Because the design of the study included only those who were alive at time of survey completion, there were no study participants with a grade 5 (fatal) condition.

CML_TKI participants’ age at CML diagnosis, sex, years between CML diagnosis and survey completion, and type of TKI were abstracted from medical records. The CML_BMT participants’ age at CML diagnosis and at BMT, sex, and BMT-related details were retrieved from institutional transplant databases and/or participants’ medical record. The Institutional Review Board (IRB) at UAB serves as the single IRB of record. Participants provided informed consent according to the Declaration of Helsinki. All methods were performed in accordance with the relevant guidelines and regulations.

The present report includes participants who completed the survey and belonged to one of the following three groups: (i) TKI-treated CML patients who did not receive a BMT (CML_TKI: enrolled between 2019 and 2023), (ii) an unaffected population (siblings of BMT survivors: non-CML controls: enrolled between 2013 and 2020), and (iii) CML patients treated with allogeneic BMT but without exposure to TKIs (CML_BMT: enrolled between 1999 and 2019). The overarching goal was to compare health status between CML_TKI patients and (i) non-CML controls and (ii) CML_BMT survivors.

### Health status measures

Four domains of health status were measured for this study: general health, functional status, activity limitations, and pre-frailty/frailty (Supplementary Table 1). Questions assessing general health, functional status, and activity limitations were adapted from the National Health Interview Survey [41] and the Behavioral Risk Factor Surveillance System Survey Questionnaire [42]. The pre-frailty/frailty phenotype was adapted [31] from the original Fried’s criteria of frailty [43].

The BMTSS survey questions used to determine self-reported health status domains are described here and in Supplementary Table 1. Self-reported general health was assessed using the following question: “*In general, would you say your health is excellent, very good, good, fair, poor?*”. For functional impairment, asked: “*Do you currently have any health problems that have resulted in: (i) needing help in personal care needs, such as eating, bathing, or getting around your home, (ii) needing help in handling routine needs, such as everyday household chores, doing necessary business, shopping, or getting around for other purposes, (iii) being unable to hold a job or attend school?”*. For activity limitation, we asked: *“Over the last 2 years, has your health limited you for more than 3 months in any of the following area: (i) vigorous activities; (ii) moderate activities like moving a table, carrying groceries, or bowling; (iii) walking uphill or climbing a few flights of stairs; (iv) bending/lifting/stooping; (v) walking one block; (vi) eating/dressing/bathing”*. A frailty phenotype was constructed from responses provided by the study participants for the following five indices: clinically underweight, exhaustion, low energy expenditure, slow walking speed, and muscle weakness. Participants were categorized as frail ( ≥ 3 indices), pre-frail (2 indices), or non-frail (0–1 indices) [31].

### Statistical analysis

Descriptive statistics were reported using mean (standard deviation [SD]), median (range), or counts (percentages) as appropriate. The prevalence of adverse outcomes in each of the health status domains was determined for the CML_TKI, non-CML, and CML_BMT cohorts. Multivariable logistic regression with each health status domain serving as the dependent variable were used to compare the odds of adverse outcomes among CML_TKI vs. (i) non-CML controls and (ii) the CML_BMT cohort, adjusting for age at survey completion, sex, annual household income, health insurance, and grade 3–4 chronic health conditions. All analyses were performed using SAS v9.4 (SAS Institute, Cary, North Carolina). Findings noted with 2-sided tests were considered statistically significant at *P* < .05.

## Results

This report includes 62 CML_TKI participants, 303 non-CML controls, and 220 CML_BMT participants. Table 1 summarizes the clinical and demographic characteristics of the study population. The median age at CML diagnosis for the CML_TKI cohort was 48.0 years (range, 16.0–73.0); the median age at survey completion was 64.0 years (range, 27.0–95.0), and the median interval between CML diagnosis and survey completion was 12.5 years (3.0–24.0). Sixty-nine percent of the CML_TKI survivors were female, and 71.0% were non-Hispanic White.

**Table 1:** Demographic characteristics and key outcomes.

	CML_TKI (*n* = 62)	Non-CML controls (*n* = 303)	*p* value (CML_TKI vs. non-CML controls)	CML_BMT (*n* = 220)	*p* value (CML_TKI vs. CML_BMT)
**Age at CML diagnosis in years**					
Median (range)	48.0 (16.0–73.0)	NA	NA	36.7 (16.7–71.1)	<0.0001
**Interval between CML diagnosis and survey completion in years**					
Median (range)	12.5 (3.0-24.0)	NA	NA	19.4 (3.0–37.9)	<0.0001
**Interval between BMT and survey completion in years**					
Median (range)	NA	NA	NA	19.0 (3.3–34.0)	__
**Age at survey completion in years**					
Median (range)	64.0 (27.0–95.0)	62.4 (27.0–90.5)	0.7397	56.0 (34.0–89.0)	<.0001
**Sex, n (%)**					
Female	43 (69.4%)	185 (61.1%)	0.2189	95 (43.2%)	0.0003
**Race/ethnicity, n (%)**					
Non-Hispanic White	44 (71.0%)	274 (90.4%)	<.0001	174 (79.1%)	<.0001
Black	13 (21.0%)	6 (2.0%)		7 (3.2%)	
Hispanic	0	15 (5.0%)		22 (10.0%)	
Other	5 (8.1%)	8 (2.6%)		17 (7.7%)	
**Education, n (%)**					
≤High school	12 (19.4%)	41 (13.5%)	0.3763	41 (18.6%)	0.9131
Some college	22 (35.5%)	96 (31.7%)		87 (39.5%)	
≥College	27 (43.5%)	164 (54.1%)		90 (40.9%)	
Missing	1 (1.6%)	2 (0.7%)		2 (0.9%)	
**Annual household income, n (%)**					
<$50,000	21 (33.9%)	56 (18.5%)	<.0001	75 (34.1%)	0.0053
$50,000-$74,999	12 (19.4%)	61 (20.1%)		35 (15.9%)	
$≥75,000	16 (25.8%)	165 (54.5%)		94 (42.7%)	
Missing	13 (21.0%)	21 (6.9%)		16 (7.3%)	
**Health insurance, n (%)**					
Private	28 (45.2%)	224 (73.9%)	<.0001	151 (68.6%)	0.0006
Public	21 (33.9%)	77 (25.4%)		53 (24.1%)	
None	13 (21.0%)	2 (0.7%)		16 (7.3%)	
**Chronic health conditions, n (%)**					
Grade 0–2	32 (51.6%)	174 (57.4%)	0.4003	64 (29.1%)	0.0009
Grade 3–4	30 (48.4%)	129 (42.6%)		156 (70.9%)	
**Poor general health, n (%)**					
No	45 (72.6%)	271 (89.4%)	<.0001	144 (57.4%)	0.7229
Yes	17 (27.4%)	15 (5.0%)		61 (24.3%)	
Missing	0	0		46 (18.3%)	
**Functional impairment, n (%)**					
No	30 (48.4%)	232 (76.6%)	<.0001	121 (55.0%)	0.3386
Yes	32 (51.6%)	70 (23.1%)		98 (44.5%)	
Missing	0	3 (0.2%)		1 (0.5%)	
**Activity limitation, n (%)**					
No	23 (37.1%)	215 (71.0%)	<.0001	96 (43.6%)	0.1767
Yes	39 (62.9%)	87 (28.7%)		109 (49.5%)	
Missing	0	0		15 (6.8%)	
**Frailty, n (%)**					
No frailty	41 (66.1%)	276 (91.1%)	<.0001	165 (75.0%)	0.2416
Pre-frailty	13 (21.0%)	22 (7.3%)		34 (15.5%)	
Frailty	8 (12.9%)	5 (1.7%)		21 (9.5%)	

*CML* denotes chronic myeloid leukemia, *TKI* denotes tyrosine kinase inhibitor, *BMT* denotes blood or marrow transplantation.

### CML_TKI survivors vs. the non-CML cohort

Compared with the non-CML cohort, the CML_TKI survivors were more likely to be non-Hispanic black (21.0% vs. 2.0%), to have a lower annual household income (<$50,000: 33.9% vs. 18.5%), and more likely to have public health insurance or no insurance (54.9% vs. 26.1%) (Table 1). However, the proportion of individuals with grade 3–4 chronic health conditions was comparable between the CML_TKI cohort and the non-CML controls (48.4% vs. 42.6%).

Table 2 compares the prevalence of individuals with impaired health status in the CML_TKI and non-CML cohorts in various demographic categories. Overall, 71.0% of CML_TKI participants had at least 1 adversely affected domain vs. 42.6% of the non-CML controls (p < 0.001). The CML_TKI participants had a statistically significantly higher prevalence of health status impairments when compared with the non-CML controls in all subgroups. The magnitude of these difference was greatest among those who were older, those with lower annual household income, those with public health insurance, and those with severe/ life-threatening chronic health conditions.

**Table 2:** Prevalence of impairments in health status in the cohorts by demographic characteristics.

	General health impairment	Functional impairment	Activity limitations	Pre-frailty or frailty	Any domain
**Age at survey** < **45 years**					
CML_TKI cohort (*n* = 9)	3 (33.3%)	3 (33.3%)	4 (44.4%)	1 (11.1%)	7 (77.8%)
Non-CML controls (n = 46)	3 (6.5%)	0	2 (4.3%)	5 (10.9%)	8 (17.4%)
CML_BMT cohort (n = 20)	6 (30.0%)	8 (40.0%)	6 (30.0%)	5 (25.0%)	11 (55.0%)
*p* value_CML_TKI vs. non-CML_	–	–	–	–	**0.0002**
*p* value_CML_TKI vs. CML_BMT_	–	–	–	–	0.2422
**Age at survey** ≥ **45 years**					
CML_TKI cohort (*n* = 53)	14 (26.4%)	29 (54.7%)	35 (66.0%)	20 (37.7%)	37 (69.8%)
Non-CML controls (*n* = 257)	12 (4.7%)	70 (27.2%)	85 (33.1%)	22 (8.6%)	121 (47.1%)
CML_BMT cohort (*n* = 200)	55 (27.5%)	90 (45.0%)	103 (51.5%)	50 (25.0%)	134 (67.0%)
* p* value_CML_TKI vs. non-CML_	**<0.0001**	**0.0001**	**<.0001**	**<.0001**	**0.0026**
* p* value_CML_TKI vs. CML_BMT_	0.7177	0.2187	0.1243	0.0654	0.6974
**Males**					
CML_TKI cohort (*n* = 19)	3 (15.8%)	8 (42.1%)	11 (57.9%)	5 (26.3%)	11 (57.9%)
Non-CML controls (*n* = 118)	5 (4.2%)	16 (13.6%)	24 (20.3%)	6 (5.1%)	34 (28.8%)
CML_BMT cohort (*n* = 125)	35 (28.0%)	48 (38.4%)	57 (45.6%)	29 (23.2%)	80 (64.0%)
*p* value_CML_TKI vs. non-CML_	–	**0.0024**	**0.0005**	**0.0016**	**0.0122**
*p* value_CML_TKI vs. CML_BMT_	–	0.7576	0.5011	0.7657	0.6072
**Females**					
CML_TKI cohort (*n* = 43)	14 (32.6%)	24 (55.8%)	28 (65.1%)	16 (37.2%)	33 (76.7%)
Non-CML controls (*n* = 185)	10 (5.4%)	54 (29.2%)	63 (34.1%)	21 (11.4%)	95 (51.4%)
CML_BMT cohort (*n* = 95)	26 (27.4%)	50 (52.6%)	52 (54.7%)	26 (27.4%)	65 (68.4%)
*p* value_CML_TKI vs. non-CML_	**<0.0001**	**0.001**	**0.0002**	**<.0001**	**0.0025**
*p* value_CML_TKI vs. CML_BMT_	0.666	0.775	0.4188	0.2446	0.3182
**Annual household income ≤$50,000**					
CML_TKI cohort (*n* = 21)	7 (33.3%)	15 (71.4%)	16 (76.2%)	9 (42.9%)	16 (76.2%)
Non-CML controls (*n* = 56)	7 (12.5%)	24 (42.9%)	26 (46.4%)	10 (17.9%)	35 (62.5%)
CML_BMT cohort (*n* = 75)	30 (40.0%)	51 (68.0%)	49 (65.3%)	31 (41.3%)	64 (85.3%)
*p* value_CML_TKI vs. non-CML_	**0.0463**	**0.0255**	**0.0195**	**0.0234**	0.2579
*p* value_CML_TKI vs. CML_BMT_	0.3808	0.7645	0.7092	0.9004	0.3204
**Annual household income $50,000–$74,999**					
CML_TKI cohort (*n* = 12)	4 (33.3%)	3 (25.0%)	8 (66.7%)	4 (33.3%)	8 (66.7%)
Non-CML controls (*n* = 61)	2 (3.3%)	10 (16.4%)	20 (32.8%)	8 (13.1%)	28 (45.9%)
CML_BMT cohort (*n* = 35)	5 (14.3%)	11 (31.4%)	18 (51.4%)	7 (20.0%)	21 (60.0%)
* p* value_CML_TKI vs. non-CML_	–	–	**0.0274**	0.0841	0.1884
* p* value_CML_TKI vs. CML_BMT_	–	–	0.4666	0.3465	0.6818
**Annual household income ≥$75,000**					
CML_TKI cohort (*n* = 16)	0	5 (31.3%)	8 (50.0%)	4 (25.0%)	9 (56.3%)
Non-CML controls (*n* = 165)	4 (2.4%)	27 (16.4%)	32 (19.4%)	9 (5.5%)	52 (31.5%)
CML_BMT cohort (*n* = 94)	22 (23.4%)	29 (30.9%)	39 (41.5%)	14 (14.9%)	52 (55.3%)
*p* value_CML_TKI vs. non-CML_	–	0.1398	**0.0051**	–	**0.0457**
*p* value_CML_TKI vs. CML_BMT_	–	0.9745	0.6472	–	0.9448
**Private Health insurance**					
CML_TKI cohort (*n* = 28)	3 (10.7%)	8 (28.6%)	13 (46.4%)	5 (17.9%)	15 (53.6%)
Non-CML controls (*n* = 224)	9 (4.0%)	37 (16.5%)	55 (24.6%)	21 (9.4%)	82 (36.6%)
CML_BMT cohort (*n* = 151)	37 (24.5%)	64 (42.4%)	69 (45.7%)	33 (21.9%)	94 (62.3%)
*p* value_CML_TKI vs. non-CML_	–	0.1193	**0.0146**	0.1642	0.082
*p* value_CML_TKI vs. CML_BMT_	–	0.171	0.7825	0.6347	0.3873
**Public health insurance**					
CML_TKI cohort (*n* = 21)	13 (61.9%)	17 (81.0%)	16 (76.2%)	9 (42.9%)	19 (90.5%)
Non-CML controls (*n* = 77)	4(5.2%)	31(40.3%)	31(40.3%)	5 (6.5%)	45 (58.4%)
CML_BMT cohort (*n* = 53)	19 (35.8%)	32 (60.4%)	35 (66.0%)	19 (35.8%)	43 (81.1%)
*p* value_CML_TKI vs. non-CML_	–	**0.0009**	**0.0035**	**<.0001**	**0.0063**
*p* value_CML_TKI vs. CML_BMT_	0.0647	0.11	0.6814	0.5752	0.3255
**No health insurance**					
CML_TKI cohort (*n* = 13)	1 (7.7%)	7 (53.8%)	10 (76.9%)	7 (53.8%)	10 (76.9%)
Non-CML controls (*n* = 2)	2 (100.0%)	2 (100.0%)	1 (50.0%)	1 (50.0%)	2 (100.0%)
CML_BMT cohort (*n* = 16)	5 (31.3%)	2 (12.5%)	5 (31.3%)	3 (18.8%)	8 (50.0%)
*p* value_CML_TKI vs. non-CML_	–	–	–	–	–
*p* value_CML_TKI vs. CML_BMT_	–	–	–	–	0.1373
**No grade 3–4 chronic health conditions**					
CML_TKI cohort (*n* = 32)	6 (18.8%)	11 (34.4%)	13 (40.6%)	6 (18.8%)	17 (53.1%)
Non-CML controls (*n* = 174)	7 (4.0%)	28 (16.1%)	38 (21.8%)	14 (8.0%)	58 (33.3%)
CML_BMT cohort (*n* = 64)	9 (14.1%)	20 (31.3%)	23 (35.9%)	6 (9.4%)	32 (50.0%)
*p* value_CML_TKI vs. non-CML_	**0.0021**	**0.016**	**0.0249**	0.0602	**0.0325**
*p* value_CML_TKI vs. CML_BMT_	0.5953	0.7576	0.7388	0.1904	0.7728
**Grade 3–4 chronic health conditions**					
CML_TKI cohort (*n* = 30)	11 (36.7%)	21 (70.0%)	26 (86.7%)	15 (50.0%)	27 (90.0%)
Non-CML controls (*n* = 129)	8 (6.2%)	42 (32.6%)	49 (38.0%)	13 (10.1%)	71 (55.0%)
CML_BMT cohort (*n* = 156)	52 (33.3%)	78 (50.0%)	86 (55.1%)	49 (31.4%)	113 (72.4%)
*p* value_CML_TKI vs. non-CML_	**<0.0001**	**0.0002**	**<.0001**	**<.0001**	**0.0004**
*p* value_CML_TKI vs. CML_BMT_	0.975	**0.0479**	**0.0057**	**0.0497**	**0.0411**

*CML* denotes chronic myeloid leukemia, *TKI* denotes tyrosine kinase inhibitor, *BMT* denotes blood or marrow transplantation.

**Fig. 1:**
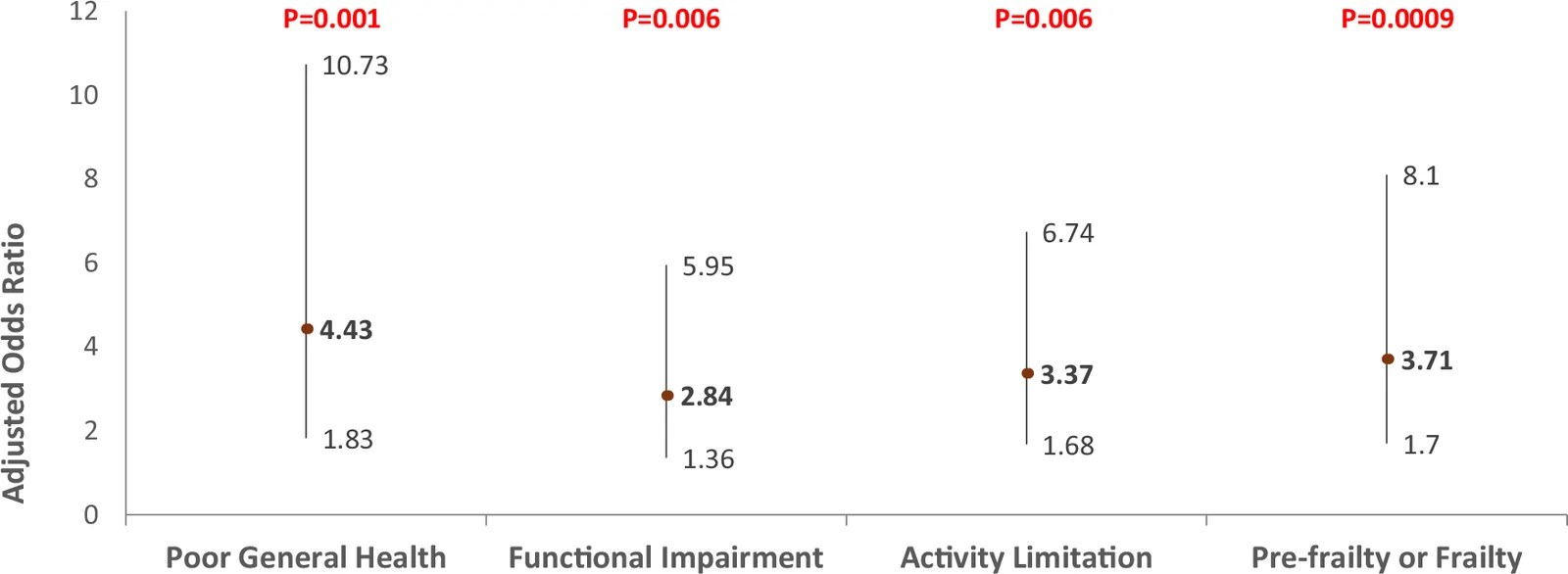
A multivariable logitsic regression analysis comparing each domain of health status between CML_TKI and non-CML controls, while adjusting for sociodemographic variables and chronic health conditions.

Figure 1 and Tables S2–S5 show the odds of adversely affected domains among CML_TKI patients vs. the non-CML cohort (presented as adjusted odds ratio [aOR] estimates with 95% confidence intervals [95%CIs]). The CML_TKI cohort was more likely to report poor general health (27.4% vs. 5.0%, aOR = 4.43, 95%CI = 1.8–10.7, *p* = 0.001), functional impairment (51.6% vs. 23.1%; aOR = 2.84, 95%CI = 1.4–6.0, *p* = 0.006), and activity limitation (62.9% vs. 28.7%; aOR = 3.37, 95%CI = 1.7–6.7, *p* = 0.0006) when compared with the non-CML cohort. The CML_TKI cohort was also more likely to be frail or pre-frail (33.9% vs. 9.0%; aOR = 3.71, 95%CI = 1.7–8.1, *p* = 0.0009) when compared with the non-CML cohort.

### CML_TKI vs. CML_BMT cohorts

As shown in Table 1, the CML_TKI cohort was older at CML diagnosis (median age: 48 years vs. 36.7 years) and at survey completion (64.0 years vs. 56.0 years) when compared with the CML_BMT cohort (Table 1), despite a shorter interval between CML diagnosis and survey completion (12.5 *vs*. 19.4 years). There was an overrepresentation of females (69.4% vs. 43.2%), non-Hispanic blacks (21.0% vs. 3.2%), and publicly insured or uninsured individuals in the CML_TKI cohort (54.9% vs. 31.4%). However, the proportion of individuals with grade 3–4 chronic health conditions was lower in the CML_TKI cohort when compared with the CML_BMT cohort (48.4% *vs*. 70.9%).

Table 2 compares the prevalence of individuals with impaired health status in the CML_TKI and CML_BMT cohorts in various demographic categories. Overall, 71.0% of CML_TKI participants had at least 1 adversely affected health status domain *vs*. 65.9% of the CML_BMT participants. There was no differences in the health status between the CML_TKI and CML_BMT subcohorts, except for individuals with severe/life-threatening chronic health conditions. In the subcohort restricted to those with individuals with severe/life-threatening chronic health conditions, the CML_TKI group was more likely to have functional impairment (70% vs. 50%), activity limitations (87% vs. 55%), and pre-frailty/frailty (50% vs. 31%). Multivariable logistic regression in this subcohort showed that the CML_TKI cohort was at a 6.7-fold greater odds of reporting activity limitations (95%CI, 1.70-26.25, p = 0.007) when compared with the CML_BMT cohort after adjusting for age, sex, income, and insurance.

**Fig. 2:**
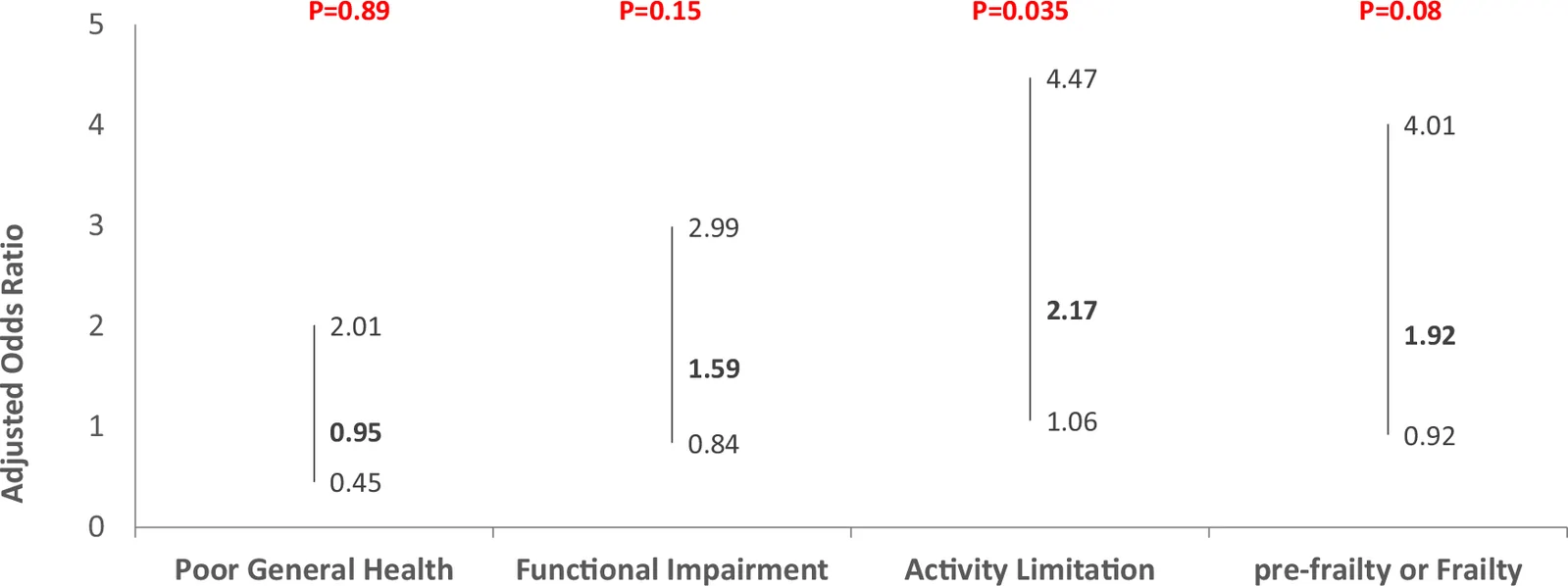
Health status in CML_TKI cs. CML_BMT cohorts.

As shown in Fig. 2 and Tables S6–S9, the CML_TKI and CML_BMT cohorts were comparable with respect to poor general health (27.4% vs. 24.3%; aOR = 0.95, 95%CI = 0.5–2.0, *p* = 0.89), functional impairment (51.6% vs. 44.5%; aOR = 1.59, 95%CI = 0.8–3.0, *p* = 0.15), and pre-frailty/frailty (33.9% vs. 25.0%; aOR = 1.92, 95%CI = 0.92–4.0, *p* = 0.08). However, the CML_TKI cohort was more likely to report activity limitation (62.9% *vs*. 49.5%, aOR = 2.2, 95%CI = 1.1–4.5, *p* = 0.035). The prevalence of activity limitation was comparable between the demographic/clinical subgroups of the CML_TKI and CML_BMT cohorts, with two notable exceptions: individuals with activity limitation were overrepresented in the CML_TKI cohort among those with grade 3–4 chronic health conditions and among those with no health insurance (Table 2). In particular, congestive heart failure was overrepresented among study participants with activity limitations in the CML_TKI cohort (11.5%) *vs*. the CML_BMT cohort (5.8%). Of note, there was no difference in the prevalence of fatigue between the CML_TKI cohort (35.5%) and the CML_BMT cohort (32.1%) (*data not shown*).

### Predictors of impaired health status in the CML_TKI cohort

As shown Table 3, presence of severe/life-threatening chronic health conditions was associated with a significantly increased odds of impaired health status. Other factors associated with a non-statistically significantly increased odds of impaired health status included older age, female sex, and public health insurance. Of note, the health status outcomes were generally comparable among patients who had received only 1^st^ generation TKIs (imatinib) vs. those who received 1st and subsequent generation TKIs or only 2nd and subsequent generation TKIs (OR = 1.41, 95%CI = 0.3–6.6).

**Table 3:** Predictors of Impaired Health Status in the CML_TKI cohort.

Risk Factors	Bivariate analysis		Multivariable analysis	
	OR (95%CI)	*p* value	OR (95%CI)	*p* value
**CML_TKI (reference:** 1**st** **generation only: n** = **24)**				
CML_otherTKI±1st generation (*n* = 38)	1.40 (0.46-4.27)	0.55	1.41(0.30–6.56)	0.66
**Age at diagnosis (reference:** < 45 **years: n** = **26)**				
≥45 years (*n* = 36)	6.20 (1.83–20.95)	0.003	3.58 (0.62–20.70)	0.16
**Sex (reference: Male: n** = **19)**				
Female (*n* = 43)	2.40 (0.76–7.61)	0.14	2.95 (0.60–14.50)	0.18
**Income (reference: ≥$75,000: n** = 16)*				
$50000–74,999 (*n* = 12)	1.56 (0.33–7.36)	0.58	1.40 (0.17–11.32)	0.75
≤$50,000 (*n* = 21)	2.49 (0.61–10.18)	0.20	0.85 (0.12–5.89)	0.87
**Education (reference: ≥College: n** = **27)**				
Some college (*n* = 22)	4.35 (1.03–18.37)	0.045	1.78 (0.28–11.33)	0.54
<=High School (*n* = 12)	1.37 (0.33–5.72)	0.66	0.17 (0.01–2.27)	0.18
**Insurance (reference: private: n** = **28)**				
Public (*n* = 21)	8.23 (1.60–42.25)	0.01	4.87 (0.45–52.25)	0.19
No insurance (*n* = 13)	2.89 (0.65–12.80)	0.16	0.88 (0.10–7.74)	0.91
**Grades 3–4 Chronic Health Conditions (reference: no: n** = **32)**				
Yes (*n* = 30)	7.94 (2.00–31.57)	0.003	16.73 (1.56–179.47)	0.02

^*^missing: *n* = 13.

*CML* denotes chronic myeloid leukemia, *TKI* denotes tyrosine kinase inhibitor, *BMT* denotes blood or marrow transplantation.

## Discussion

The current report examined the difference in the health status reported by TKI-treated CML patients and two comparison groups: an unaffected population, and CML patients treated with BMT but without exposure to TKIs. After a median of 12 years from CML diagnosis, the TKI-treated CML patients were at a threefold to fourfold greater odds of reporting poor general health, functional impairment, activity limitation, and pre-frailty/frailty when compared with the unaffected comparison group. In contrast, the TKI-treated group did not differ from the CML patients treated with BMT with respect to poor general health, functional impairment, or pre-frailty/frailty. However, the TKI-treated CML patients were at a twofold greater odds of activity limitation when compared with the BMT survivors.

Previous studies have focused on disease recurrence, toxicities, and overall survival among TKI-treated CML patients [7–24]. Pooled data from multiple frontline TKI-based trials have reported excellent survival [44]. However, using real-world data, Chow *et al* reported that TKI-treated CML patients had a significantly greater burden of adverse events across most organ system categories compared with the general non-cancer population [9]. These differences were apparent within the first year of treatment and persisted through 5 years.

A scoping review of CML patients receiving TKIs has demonstrated impaired overall health-related quality of life (HRQoL) when compared with the general population [45]. The patient-reported outcome (PRO) measures used to study HRQoL have included the SF-36, FACT-Leu, EORTC QLQ-CML24, and MDASI CML. The outcomes have been measured in patients who received TKIs for an average of 4–5 y [46–50] as well as at 12 months after discontinuation of TKIs [51]. The current study differs from these previous reports in focusing on a multidimensional approach to the self-report of health status comprising of impairments in general health, functional impairment, activity limitation, and frailty. Furthermore, no previous studies have described the health status of CML patients receiving prolonged TKI treatment and compared these outcomes with non-TKI exposed BMT survivors. In particular, previous studies were unable to adjust for key sociodemographic factors such as annual household income and health insurance coverage or for severe/life-threatening comorbidity.

In the present study, we defined health status of CML patients treated with TKIs for a median of 12 years in the context of four domains that assessed general health, functional impairment, activity limitations, and pre-frailty or frailty. Self-rated general health is an independent predictor of mortality [26–29]. Functional impairment and activity limitations represent an inability to perform everyday tasks are a key indicator of overall health, signaling potential risks for falls, social isolation, hospitalization, and nursing home admission [30]. Both pre-frailty and frailty are associated with a higher hazard of mortality in both non-cancer populations [32, 33] and among cancer survivors [31, 34]. Over 70% of the CML_TKI participants had at least one adversely affected domain; in comparison, 43% of the non-CML controls were adversely affected. Adjusting for sociodemographics and comorbidity, the CML_TKI cohort was at a 2.8-fold to 4.4-fold greater odds of experiencing poor health status when compared with the non-CML cohort, suggesting the role of the underlying disease and/or treatment effects in determining health status. In contrast, the health status of the CML_TKI cohort was comparable to the CML_BMT cohort, with the exception of a two-fold greater odds of adverse activity limitations after adjusting for socio-demographics and comorbidities.

Fatigue is a prevalent and debilitating side effect in CML patients receiving TKI treatment; the prevalence of severe fatigue was reported as 55.5% in CML patients and 10.9% in controls in a previous study [52]. Objective monitoring of activity patterns confirmed that fatigued CML patients performed less light and moderately intensity physical activity and worked fewer hours when compared to non-fatigued patients. The prevalence of fatigue among the CML_TKI individuals in our study was 35.5% and was comparable to the prevalence of fatigue among the CML patients treated with BMT (32.1%), suggesting that the greater odds of activity limitation in the CML_TKI patients could not be explained by fatigue.

However, the CML_TKI patients with activity limitations were twice as likely to have congestive heart failure as compared to the CML_BMT patients with activity limitations, suggesting that higher prevalence of CHF in the CML_TKI participants (a known complication [53]) likely contributed to the greater odds of activity limitations.

An increased risk of infections, cardiopulmonary and skin issues have been reported with later generations of TKIs when compared with imatinib [9, 10, 24]. In the current report we found that patients who had received only first generation TKIs were generally comparable with those who received first and subsequent generation TKIs or only 2^nd^ and subsequent generation TKIs with respect to impairment in one or more of the health status domains, after adjusting for severe/ life-threatening comorbidity.

These findings are relevant to patient priorities regarding treatment goals and quality of life. A survey of 582 patients with CML in Germany (median age of 56 years and median duration of TKI treatment >5 years), indicated substantial heterogeneity in the acceptance of adverse treatment-related effects. Younger patients prioritized the preservation of fertility, sexual health, and occupational activity, whereas older patients ( > 65 years) prioritized mental health, independence in daily tasks, and tolerability of therapy [54]. Long-term survival, prevention of relapse, and maintenance of quality of life were the most highly prioritized treatment goals across the cohort consistent with a previous survey [55]. The international CML SUN Survey showed that in general healthcare providers placed greater emphasis on treatment efficacy, whereas patients prioritized quality of life and tolerability, while maintaining disease control [56]. A Danish nationwide cohort study demonstrated that many CML patients struggle to return to the work despite successful disease control, especially older individuals, women, and those with longer disease duration [57]. An expert consensus panel recently defined TKI treatment intolerance in terms of adverse effects that interfered with daily activities, often leading to TKI modifications [58]. Interestingly, while discontinuation of TKIs has been associated with improvements in patient-reported outcomes (PROs) [59], this was not always the case; in the EuroSKI trial younger patients were more likely to report improvements in function and symptoms after discontinuing TKIs, whereas patients aged 60 years or above showed negligible benefit [60].

Findings from this study need to be placed in the context of its limitations. Health status was determined by self-report and not validated externally. We did not use a single validated instrument for assessment of health status but derived this measure from a combination of various instruments. Furthermore, the fact that we did not use CML-specific measures could have limited the ability to capture more nuanced aspects of the health status in this population. We did not have information on the pre-treatment health status. We were limited by a relatively small convenience sample size of the CML_TKI cohort, compromising the ability to conduct a comprehensive evaluation of the health status within each of the sociodemographic subgroups or by exposure to different generations of TKIs. These limitations notwithstanding, this is the first comparison of the health status reported by CML patients treated with TKIs for a median of 12 years with a non-cancer cohort and non-TKI exposed CML cohort treated with BMT. Findings from this study suggest a need for close monitoring of the health status of TKI-treated CML patients and development of strategies for risk mitigation as a treatment goal in CML care.

## Supplementary information

Supplemental Material

## Data Availability

The data sets generated during and/or analyzed during the current study are not publicly available but are available from the corresponding author on reasonable request.
