# Supplementary material for: Health status impairments in tyrosine kinase inhibitor-treated chronic myeloid leukemia patients versus blood or marrow transplant survivors and non-cancer controls
Source: Leukemia. 2026 Aug 21;40(11):2494–502. doi: 10.1038/s41375-026-03123-w (PMC13713547; doi:10.1038/s41375-026-03123-w)
Supplement: Supplementary file 1 [file 41375_2026_3123_MOESM1_ESM.docx]

**Supplement**

**Health Status of Chronic Myeloid Leukemia patients treated with Tyrosine Kinase Inhibitors**

**Bhatia *et. al.***

**Poor General Health**

*“Would you say that your current health is…”*

***Answers:*** Excellent, Very good, Good, Poor, Fair

***Poor General Health:*** Poor or Fair

**Functional Impairment**

*“Do you currently have any health problems that have resulted in…”*

- Needing help with personal care needs, such as bathing, dressing, or getting around your home
- Needing help in handling routine needs, such as everyday household chores, shopping or getting around for other purposes

Being unable to hold a job or attend school

***Functional Impairment:*** Answer “yes” to any of the above questions

**Activity Limitation**

*“Over the last 2 years, has your health limited you for more than 3 months in any of the following areas…”*

- The kinds or amounts of vigorous activities you can do, like lifting heavy objects, running, or participating in strenuous sports
- The kinds or amounts of moderate activities you can do, like moving a table, carrying groceries, or bowling
- Walking uphill or climbing a few flights of stairs
- Bending, lifting, or stooping
- Walking one block
- Eating, dressing, bathing, or using the toilet

***Activity Limitation***: Answer of “yes” to ≥3 of the above questions

**Frailty**

- **Weight loss/ undernourished:** BMI <18.5
- **Exhaustion** *“How much of the time during the past 4 weeks. did you feel: a) worn out? (b) tired?”*
  a. ***6=none of the time; 5=a little of the time, 4= some of the time; 3=a good bit of the time, 2=most of the time, 1=all of the time.*** Subjects answering <4 to either question are categorized as frail by exhaustion criterion
- **Physical activity/ energy expenditure:** ***“****How much does your health limit you in running, lifting heavy objects, participating in sports?”*
  a. ***1=no, not limited at all; 2=yes, limited a little; 3=yes, limited a lot.*** “3” = frail by the physical activity criterion
- **Walk performance/ slowness:** *“Does your health limit you in climbing 1 flight of stairs/ walking 1 block?”*
  a. ***1=not limited; 2=limited a little; 3=limited a lot.*** Subjects answering “3” to either question categorized as frail by the “slowness” criterion
- **Physical strength/ weakness:** “How much has feeling weak in parts of your body distressed/bothered you during the past 7 days including today?”
- ***0=Not at all 1=A little bit, 2=Moderately/Quite a bit, 3=Extremely.*** Subjects answering “3” to this question are categorized as frail by the weakness criterion

**Frail:** presence of ≥3 indices

**Pre-frail:** presence of 2 indices

**Non-frail:** presence of 0-1 indices

**Table S1. Definition of health domains**

**Table S2. Poor General Health in CML_TKI vs. non-CML controls**

| **Risk Factors** | **Bivariate analysis** | | **Multivariable analysis** | |
| --- | --- | --- | --- | --- |
|  | **OR (95%CI)** | **p-value** | **OR (95%CI)** | **p-value** |
| **CML_TKI vs. non-CML (reference: non-CML controls)** | | | | |
| CML_TKI | 6.8 3(3.18-14.63) | <.0001 | 4.43 (1.83-10.73) | 0.0010 |
| **Age at study (reference: <45 years)** | | | | |
| ≥45 | 0.80 (0.31-2.03) | 0.6322 | 0.51 (0.17-1.59) | 0.2476 |
| **Sex (reference: Male)** | | | | |
| Female | 1.94 (0.84-4.45) | 0.1190 | 1.67 (0.67-4.15) | 0.2700 |
| **Income (reference:** **≥$75,000)** | | | | |
| $50,000-74,999 | 3.94 (1.08-14.41) | 0.0384 | 3.40 (0.90-12.84) | 0.0712 |
| ≤$50,000 | 9.80 (3.10-30.94) | <.0001 | 5.35 (1.56-18.28) | 0.0075 |
| **Insurance (reference: Private)** | | | | |
| Public | 4.33 (1.98-9.47) | 0.0002 | 2.34 (0.93-5.89) | 0.0707 |
| No | 4.77 (1.19-19.20) | 0.0277 | 0.89 (0.18-4.51) | 0.8919 |
| **Grades 3-4 Chronic Health Conditions (reference: no)** | | | | |
| Yes | 2.15 (1.02-4.50) | 0.0431 | 1.9 1(0.81-4.48) | 0.1378 |

**Table S3. Functional Impairment in CML_TKI vs. non-CML controls**

| **Risk Factors** | **Bivariate analysis** | | **Multivariableanalysis** | |
| --- | --- | --- | --- | --- |
|  | **OR (95%CI)** | **p-value** | **OR (95%CI)** | **p-value** |
| **CML_TKI vs. non-CML controls (reference: non-CML controls)** | | | | |
| CML_TKI | 3.54 (2.01-6.22) | <.0001 | 2.84 (1.36-5.95) | 0.0055 |
| **Age at study (reference: <45 years)** | | | | |
| ≥45 | 8.17 (2.49-26.80) | 0.0005 | 9.37 (2.45-35.85) | 0.0011 |
| **Sex (reference: Male)** | | | | |
| Female | 2.46 (1.47-4.14) | 0.0007 | 2.83 (1.55-5.17) | 0.0007 |
| **Income (reference:** ≥$75,000) | | | | |
| $50000-74,999 | 1.00 (0.49-2.04) | 0.9954 | 0.86 (0.40-1.87) | 0.7035 |
| ≤$50,000 | 4.75 (2.64-8.54) | <.00 | 2.87 (1.47-5.60) | 0.0021 |
| **Insurance (reference: Private)** | | | | |
| Public | 4.39 (2.64-7.32) | <.0001 | 2.81 (1.56-5.05) | 0.0006 |
| No | 6.87 (2.33-20.26) | 0.0005 | 1.54 (0.41-5.79) | 0.5209 |
| **Grades 3-4 Chronic health conditions (reference: no)** | | | | |
| Yes | 2.79 (1.74-4.48) | <.0001 | 2.38 (1.37-4.12) | 0.0020 |

**Table S4. Activity Limitation in CML_TKI vs. non-CML controls**

| **Risk Factors** | **Bivariate analysis** | | **Multivariable analysis** | |
| --- | --- | --- | --- | --- |
|  | **OR (95%CI)** | **p-value** | **OR (95%CI)** | **p-value** |
| **CML_TKI vs. non-CML controls (reference: non-CML controls)** | | | | |
| CML_TKI | 4.19 (2.36-7.43) | <.0001 | 3.37 (1.68-6.74) | 0.0006 |
| **Age at study (reference: <45 years)** | | | | |
| ≥45 | 5.19 (2.15-12.48) | 0.0002 | 4.80 (1.82-12.68) | 0.0015 |
| **Sex (reference: Male)** | | | | |
| Female | 1.95 (1.22-3.11) | 0.0051 | 1.97 (1.17-3.33) | 0.0111 |
| **Income (reference:** ≥$75,000) | | | | |
| $50000-74,999 | 2.18 (1.21-3.92) | 0.0095 | 2.27 (1.19-4.34) | 0.013 |
| ≤$50,000 | 4.20 (2.38-7.43) | <.0001 | 2.82 (1.49-5.34) | 0.0015 |
| **Insurance (reference: Private)** | | | | |
| Public | 2.48 (1.53-4.03) | 0.0002 | 1.60 (0.92-2.81) | 0.0974 |
| No | 7.40 (2.28-24.03) | 0.0009 | 1.93 (0.49-7.55) | 0.3465 |
| **Grades 3-4 Chronic health conditions (reference: no)** | | | | |
| Yes | 2.70 (1.73-4.20) | <.0001 | 2.35 (1.43-3.88) | 0.0008 |

**Table S5. Pre-frailty or/and frailty in CML_TKI vs. non-CML controls**

| **Risk Factors for pre-Frailty** | **Bivariate analysis** | | **Multivariable analysis** | |
| --- | --- | --- | --- | --- |
|  | **OR (95%CI)** | **p-value** | **OR (95%CI)** | **p-value** |
| **CML_TKI vs. non-CML (reference: non-CML controls)** | | | | |
| CML_TKI | 5.24 (2.71-10.11) | <.0001 | \| 3.71 (1.71-8.07) \| | 0.0009 |
| **Age at study (reference: <45 years)** | | | | |
| ≥45 | 1.28 (0.52-3.17) | 0.59 | 0.97 (0.35-2.69) | 0.96 |
| **Sex (reference: Male)** | | | | |
| Female | 2.22 (1.09-4.51) | 0.03 | 2.00 (0.94-4.26) | 0.07 |
| **Income (reference:** **≥$75,000)** | | | | |
| $50000-74,999 | 2.54 (1.10-5.87) | 0.03 | 2.50 (1.03-6.05) | 0.04 |
| ≤$50,000 | 4.23 (1.97-9.11) | 0.0002 | 2.87 (1.22-6.77) | 0.02 |
| **Insurance (reference: Private)** | | | | |
| Public | 1.45 (0.72-2.91) | 0.30 | 0.99 (0.45-2.20) | 0.99 |
| No | 9.93 (3.33-29.62) | <.0001 | 3.19 (0.87-11.77) | 0.08 |
| **Grades 3-4 Chronic health conditions (reference: no)** | | | | |
| Yes | 1.99 (1.07-3.68) | 0.03 | 1.85 (0.93-3.70) | 0.08 |

**Table S6. Poor General Health in CML_TKI vs CML_BMT cohorts**

| **Risk Factors** | **Bivariate analysis** | | **Multivariable analysis** | |
| --- | --- | --- | --- | --- |
|  | **OR (95%CI)** | **p-value** | **OR (95%CI)** | **p-value** |
| **CML_TKI vs. CML_BMT** | | | | |
| CML_TKI | 0.89 (0.47-1.68) | 0.7230 | 0.95 (0.45-2.01) | 0.8944 |
| **Age at study (reference: <45 years)** | | | | |
| ≥45 | 0.66 (0.28-1.58) | 0.3519 | 0.42 (0.16-1.11) | 0.0816 |
| **Sex (reference: Male)** | | | | |
| Female | 1.09 (0.64-1.84) | 0.7577 | 0.94 (0.52-1.70) | 0.8391 |
| **Income (reference:** **≥$75,000)** | | | | |
| $50000-74,999 | 0.93 (0.39-2.22) | 0.8734 | 0.92 (0.37-2.30) | 0.8657 |
| ≤$50,000 | 2.65 (1.41-4.99) | 0.0025 | 2.13 (1.03-4.42) | 0.0416 |
| **Insurance (reference: Private)** | | | | |
| Public | 2.61 (1.45-4.69) | 0.0014 | 2.04 (1.04-3.99) | 0.038 |
| No | 0.83 (0.32-2.18) | 0.7023 | 0.66 (0.23-1.88) | 0.4363 |
| **Grades 3-4 Chronic health conditions (reference: no)** | | | | |
| Yes | 3.02 (1.60-5.68) | 0.0006 | 3.06 (1.55-6.05) | 0.0012 |

**Table S7. Functional Impairment in CML_TKI vs. CML_BMT cohorts**

| **Risk Factors** | **Bivariate analysis** | | **Multivariable analysis** | |
| --- | --- | --- | --- | --- |
|  | **OR (95%CI)** | **p-value** | **OR (95%CI)** | **p-value** |
| **CML_TKI vs. CML_BMT** | | | | |
| CML_TKI | 1.32 (0.75-2.32) | 0.3393 | 1.59 (0.84-2.99) | 0.1529 |
| **Age at study (reference: <45 years)** | | | | |
| ≥45 | 1.46 (0.66-3.23) | 0.3441 | 1.03 (0.48-2.21) | 0.931 |
| **Sex (reference: Male)** | | | | |
| Female | 1.85 (1.15-2.97) | 0.0113 | 1.15 (0.70-1.91) | 0.5803 |
| **Income (reference**: **≥$75,000)** | | | | |
| $50000-74,999 | 0.98 (0.46-2.06) | 0.9533 | 1.11 (0.54-2.27) | 0.7756 |
| ≤$50,000 | 4.92 (2.72-8.88) | <.0001 | 4.42 (2.36-8.27) | <.0001 |
| **Insurance (reference: Private)** | | | | |
| Public | 3.03 (1.71-5.38) | 0.0001 | 1.50 (0.81-2.78) | 0.1973 |
| No | 0.67 (0.29-1.55) | 0.3490 | 0.37 (0.15-0.92) | 0.0313 |
| **Grades 3-4 Chronic health conditions (reference: no)** | | | | |
| Yes | 2.41 (1.44-4.04) | 0.0008 | 2.63 (1.54-4.50) | 0.0004 |

**Table S8. Activity Limitation in CML_TKI vs. CML_BMT cohorts**

| **Risk Factors** | **Bivariate analysis** | | **Multivariable analysis** | |
| --- | --- | --- | --- | --- |
|  | **OR (95%CI)** | **p-value** | **OR (95%CI)** | **p-value** |
| **BMT vs. TKI** | | | | |
| TKI | 1.49 (0.83-2.68) | 0.1781 | 2.17 (1.06-4.47) | 0.035 |
| **Age at study (reference: <45 years)** | | | | |
| ≥45 | 1.84 (0.79-4.31) | 0.1599 | 1.55 (0.60-3.97) | 0.3624 |
| **Sex (reference: Male)** | | | | |
| Female | 1.47 (0.90-2.38) | 0.1227 | 1.37 (0.78-2.41) | 0.2729 |
| **Income (reference:** **≥$75,000)** | | | | |
| $50000-74,999 | 1.69 (0.83-3.42) | 0.1456 | 1.64 (0.76-3.52) | 0.2035 |
| ≤$50,000 | 3.34 (1.82-6.13) | <.0001 | 2.25 (1.13-4.47) | 0.0214 |
| **Insurance (reference: Private)** | | | | |
| Public | 2.82 (1.53-5.17) | 0.0008 | 2.24 (1.11-4.52) | 0.0239 |
| No | 1.12 (0.51-2.47) | 0.7720 | 0.83 (0.33-2.09) | 0.6953 |
| **Grades 3-4 Chronic health conditions (reference: no)** | | | | |
| Yes | 2.96 (1.76-4.97) | <.0001 | 3.46 (1.93-6.20) | <.0001 |

**Table S9. Pre-frailty or frailty in CML_TKI vs. CML_BMT cohorts**

| **Risk Factors** | **Bivariate analysis** | | **Multivariable analysis** | |
| --- | --- | --- | --- | --- |
|  | **OR (95%CI)** | **p-value** | **OR (95%CI)** | **p-value** |
| **BMT survivors vs. Cancer control** | | | | |
| Cancer control | 1.54 (0.84-2.82) | 0.1661 | 1.92 (0.92-4.01) | 0.0833 |
| **Age at study (reference: <45 years)** | | | | |
| ≥45 | 1.47 (0.57-3.75) | 0.4250 | 1.25 (0.45-3.44) | 0.6698 |
| **Sex (reference: Male)** | | | | |
| Female | 1.42 (0.83-2.40) | 0.1976 | 1.21 (0.67-2.18) | 0.5342 |
| **Income (reference:** **≥$75,000)** | | | | |
| $50000-74,999 | 1.56 (0.67-3.63) | 0.3001 | 1.46 (0.60-3.54) | 0.4002 |
| ≤$50,000 | 3.65 (1.91-6.98) | <.0001 | 2.62 (1.26-5.43) | 0.0099 |
| **Insurance (reference: Private)** | | | | |
| Public | 2.26 (1.25-4.08) | 0.0069 | 1.50 (0.76-2.96) | 0.2436 |
| No | 1.95 (0.84-4.55) | 0.1207 | 1.37 (0.53-3.57) | 0.5173 |
| **Grades 3-4 Chronic health conditions (reference: no)** | | | | |
| Yes | 3.67 (1.87-7.22) | 0.0002 | 3.97 (1.92-8.20) | 0.0002 |
